# Implementation of a Virtual Cardiology Curriculum to Address the Deficit of Cardiovascular Education in Haiti

**DOI:** 10.1016/j.jacadv.2024.101380

**Published:** 2024-12-26

**Authors:** Cosmas Sibindi, Veathyelau Saint-Joy, Michel Ibrahim, Kezia Domond, Ann Tierney, Berenice Loubeau, Dawson Calixte, Norrisa Haynes

**Affiliations:** a Yale School of Medicine, New Haven, Connecticut, USA; b LA BASSE -TERRE HOSPITAL CENTRE, Basse Terre (Guadeloupe), Basse-Terre, Guadeloupe; c ChenMed, Miami, Florida, USA; d Center for Global Health MGH, Boston, Massachusetts, USA; e Center for Clincal Epidemiology and Biostatics, University of Pennsylvania, Philadelphia, Pennsylvania, USA; f Centro de Diagnóstico Medicina Avanzada y Telemedicina, Santo Domingo, Dominican Republic

**Keywords:** global cardiovascular health, Haiti, implementation science

## Abstract

**Background:**

Cardiovascular disease (CVD) is the leading cause of death in low- and middle-income countries such as Haiti. Our team has demonstrated in a pilot study the implementation of a virtual cardiology curriculum to address the deficit of cardiology education in Haiti among medicine residents.

**Objectives:**

The objective of this study was to determine if cardiology education can be delivered nationwide in Haiti via a virtual platform with quantifiable improvement.

**Methods:**

Over 1 academic year, we recruited internal medicine residents from all residency years and all 4 of the internal medicine training programs in Haiti. They were enrolled in a trimester curriculum of biweekly, synchronous and asynchronous didactic lectures, seminars, and case presentations delivered via an interactive virtual classroom. Pre-trimester and post-trimester assessments were delivered to the students. Knowledge acquisition was analyzed by way of Cohen’s *r* effect sizes with 0.1 to <0.3 interpreted as small, 0.3 to <0.5 as moderate, and >0.5 as large.

**Results:**

A total of 62 residents were enrolled, 26 in their first year, 21 in their second, and 15 in their third year. There was significant improvement in CVD knowledge with all residency classes showing moderate to large effect sizes. There were notable differences in the effect sizes for residency programs in different locations. There was also significant student attrition over time likely due, in part, to political instability.

**Conclusions:**

This study demonstrates that it is possible to virtually deliver cardiology education to trainees in low- and middle-income countries on a national scale to address the deficit of CVD education. Aside from uncontrollable factors like political instability, attrition can be improved by formalization of the curriculum.

Cardiovascular disease (CVD) has emerged as a leading cause of death in low- and middle-income countries (LMICs), surpassing communicable diseases.^1^^,^^2^ This shift is attributable to successful global efforts in managing communicable diseases such as HIV, TB, and malaria.^3^ However, LMICs are often ill-equipped to tackle the growing CVD epidemic due to limited resources, infrastructure, health care workers, and medical professionals.

Haiti is a notable example of an LMIC grappling with these challenges. It remains among the poorest countries in the Latin America and Caribbean region and the world.^3^^,^^4^ With approximately 59 percent of its population living below the poverty line, access to essential services such as clean water and health care is severely limited. Haiti’s health care system is further strained by sociopolitical instability, gang violence, natural disasters, the COVID-19 pandemic, and ongoing political turmoil.^3^ These challenges have caused a significant number of Haitian medical professionals to emigrate in search of better opportunities.

Currently, up to 45% of internal medicine admissions in Haiti are related to cardiac morbidity, including heart failure.^5^ Additionally, CVD accounts for at least 29% of all adult deaths, with nearly 1 in 3 adults in Port-au-Prince living with hypertension.^6^^,^^7^ The impact of CVD in Haiti extends into maternal and child health, with 1 in every 300 pregnancies resulting in postpartum cardiomyopathy, one of the highest incidences in the world.^8^^,^^9^ Moreover, because of Haiti’s age pyramid structure, there is proportionally more morbidity from congenital heart disease as well as rheumatic disease in children. Congenital heart disease in Haiti accounts for more than double the world average national disability-adjusted life years at 2.15% compared to 0.74%. Rheumatic heart disease, on the other hand, is associated with 50% more disability-adjusted life years in Haiti at 0.66% compared to the world average of 0.42%.^10^

Despite the high prevalence of CVD, as of 2020, Haiti has only 16 cardiologists serving a population of over 11 million people.^10^ Furthermore, Haiti lacks a domestic cardiology training program, requiring cardiologists to train abroad. While most hospitals are equipped with electrocardiograms, the availability of cardiac ultrasound is limited, and the entire country lacks a catheterization lab. This scarcity in cardiology expertise forces internal medicine trainees and practitioners to manage complex cardiac conditions with inadequate training and supervision.

The Global Medical Education Network, a nonprofit organization, was established to build cardiovascular capacity in LMICs through medical education, aiming to improve cardiovascular health care and address inequalities in Haiti. In 2019 to 2020, we successfully implemented a pilot virtual cardiology curriculum, the International Cardiology Curriculum Accessible by Remote Distance Learning at Hôpital Universitaire de Mirebalais (HUM) in Haiti.^11^ This study aims to scale the project nationwide, hypothesizing that virtual cardiology education can be effectively delivered across Haiti, leading to measurable improvements in medical education and health care outcomes.

## Methods

### Site selection

**Figure 1:**
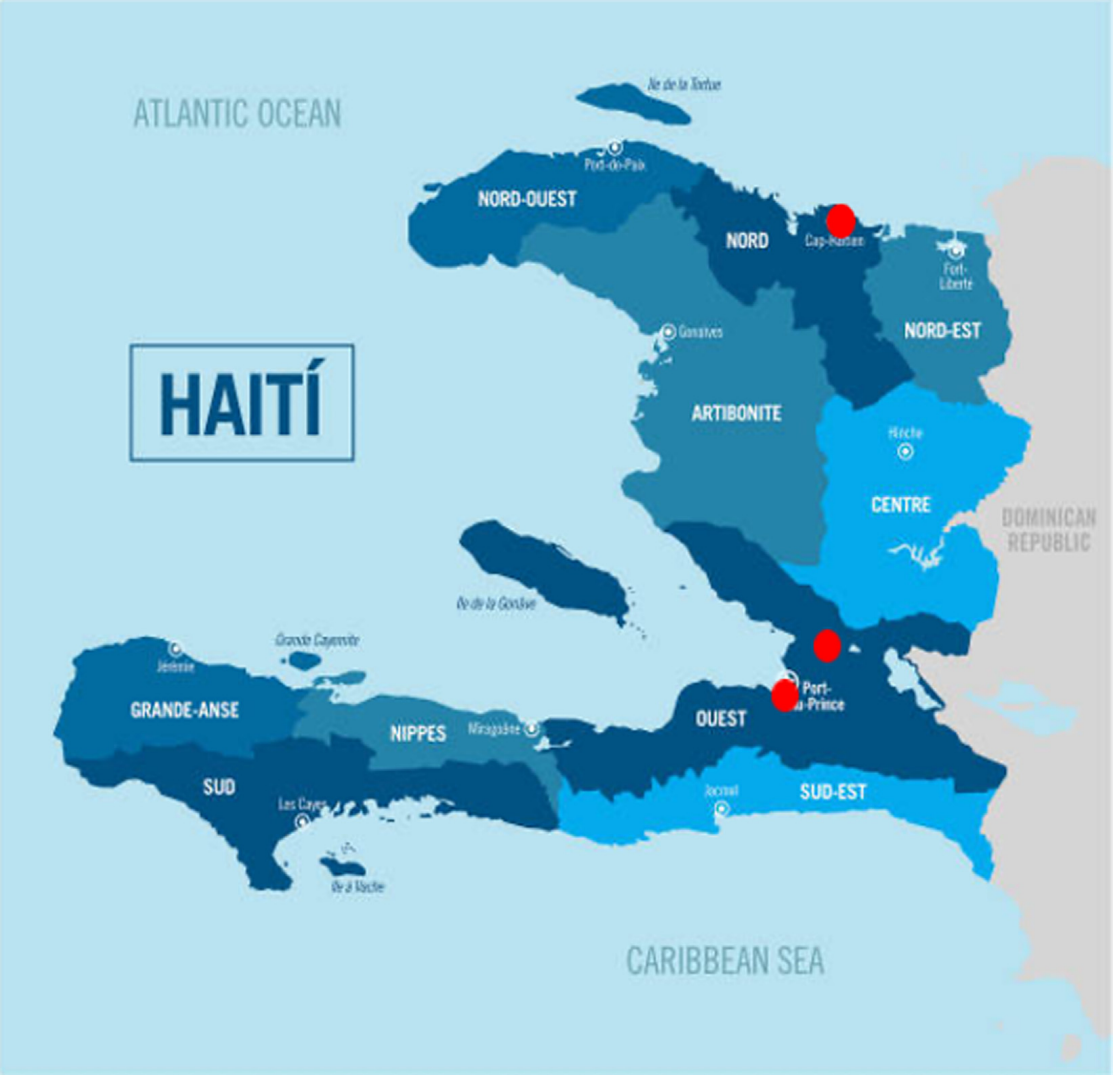
Location of Hospital Sites in Haiti

In addition to the HUM, Haiti has 3 additional teaching hospitals: General Hôpital, Hôpital Universitaire Justinien (HUJ), and Hôpital Universitaire de la Paix (La Paix). The locations of the 4 training programs are included in Figure 1. La Paix and General Hôpital are in Port-au-Prince, HUM is located in a rural community North of Port-au-Prince, and HUJ is located in the northern city of Cap-Haïtien. Together, the 4 hospitals have a total of 144 internal medicine residents. The study was conducted from January 2021 to August 2021.

The expansion of the International Cardiology Curriculum Accessible by Remote Distance Learning-Haiti beyond HUM represents a longitudinal, quasi-experimental study intended to measure the efficacy and reach of a virtual education model across multiple health care settings in Haiti.^11^

### Participant selection

The program was expanded to include a broader demographic within the Haitian medical community, explicitly selecting first- to third-year residents at 4 major hospitals across the country during the 2020 to 2021 academic year. The hospitals represent vastly different regions of Haiti, ranging from rural to densely populated urban centers. First- and second-year residents were required to participate due to their formative stage in medical education, with optional participation extended to third-year residents.

### Curriculum development and delivery

The curriculum consisted of biweekly live-streamed, interactive sessions including foundational knowledge and case-based learning, specifically tailored to the typical cardiac scenarios encountered in Haitian hospitals. Lecture content was created by a coalition of Haitian faculty educators and cardiology fellows from internationally recognized institutions like the University of Pennsylvania, SUNY Downstate, Boston University, the University of Miami, and Columbia, utilizing the American College of Cardiology’s core competency recommendations. Lectures were primarily delivered in Haitian Creole and French to maximize understanding and retention, with materials available in French and English for supplementary review.

Educational materials and lectures were facilitated using commercially available videoconferencing and data-sharing platforms, enabling simultaneous access to all enrolled hospitals. This method ensured consistent educational delivery and facilitated real-time interaction between students and educators. A dedicated educational virtual discussion board was developed and integrated into the education delivery platform. This allowed trainees to share clinical cases, post questions, or comments to which their peers and instructors could respond.

### Instructional design

Consistent with the pilot, the curriculum employed the Analysis, Design, Development, Implementation, and Evaluation model for continuous improvement based on direct feedback from participants.^11^ Knowledge assessment was conducted through pre- and post-assessments both before and after each 3 to 4 month trimester to evaluate longer-term knowledge retention. A broad spectrum of topics was covered, as outlined in Supplemental Table 1. The trimesters will be referred to as units in the rest of the text, ranging from unit 1 to unit 3.

Lecturers were provided with learning objectives derived from needs assessments, which were based on discussions with local physicians, including cardiologists, and recommendations from the American College of Cardiology. Additionally, they were tasked with formulating questions aligned with their lecture content for the pre- and post-unit assessments. Before implementation, an assessment committee meticulously reviewed these questions for appropriateness, relevance, and accuracy.

### Evaluation metrics and statistical analysis

To quantitatively measure the curriculum’s impact, effect sizes were calculated from knowledge assessments conducted before and after each unit. These assessments employed a combination of multiple-choice questions, case analysis, and simulated patient interactions to comprehensively evaluate both theoretical knowledge and practical application.

Wilcoxon signed rank exact tests were calculated on the pretest to posttest change in scores. Cohen’s *r* effect sizes (Z/square root N) were used to quantify the educational impact, with thresholds set for small (0.1 to <0.30), moderate (0.3 to <0.5), and large (≥0.5) effect sizes. Data analysis was performed using SAS (SAS Institute), 9.4 similar to the pilot study.

### Regulatory considerations

Ethical clearance was obtained from the local Haitian hospitals’ ethics committees and the University of Pennsylvania’s Institutional Review Board, ensuring compliance with both local regulations and international ethical standards. Informed consent was obtained from all participants, who were assured of their right to withdraw from the study at any time without penalty.

### Program evaluation

In-depth feedback was solicited via structured surveys and informal focus groups to capture participants’ qualitative evaluations of the curriculum. These assessments focused on educational satisfaction, relevance to clinical practice, and logistical execution. This feedback was crucial for ongoing curriculum refinements and highlighted additional training needs. The qualitative methodology was conducted by consultants at the University of Pennsylvania Mixed Methods Research Lab.

### Cost, sustainability, and future directions

In the future, the study aims to incorporate a sustainability plan that involves training and gradually transitioning curriculum leadership to Haitian instructors through certification. Additionally, through the study, a library of recorded lectures and assessments has been created and will be accessible to the training programs in perpetuity. This approach aims to build local capacity and ensure the program’s long-term viability beyond the scope of the study. This detailed methodological framework supports the objective of scaling an effective cardiology education program across Haiti through a robust, adaptable virtual learning model. This approach aims not only to elevate the standard of medical education but also to create a sustainable blueprint for other specialties and regions facing similar educational challenges. Initial investments for this program were focused on the foundational cost of hosting the online platform and the videoconferencing platform which was funded by small institutional grants. Beyond these initial costs, faculty from Haiti and around the world volunteered their time to generate the curriculum which is now housed in a virtual library available to Haitian institutions and colleagues. Ongoing efforts through Global MedEd are working to ensure that the 4 training programs which were involved in this national program can each then administer the curriculum to their residents. Additionally, we envision a continuing medical education course in the future. These efforts will require little additional funding and will be embedded in current structures.

## Results

### Demographics and class participation

From August 2021 to June 2022, a total of 62 residents enrolled across the 4 internal medicine residency training programs. Gender breakdown data were not available. Enrollment numbers were recorded at the start of each unit, with 62 residents initially enrolled who then went on to watch and complete 80% of the content. Unit 1 began with 62 residents, unit 2 began with 52 residents, and unit 3 began with 32 residents.

**Figure 2:**
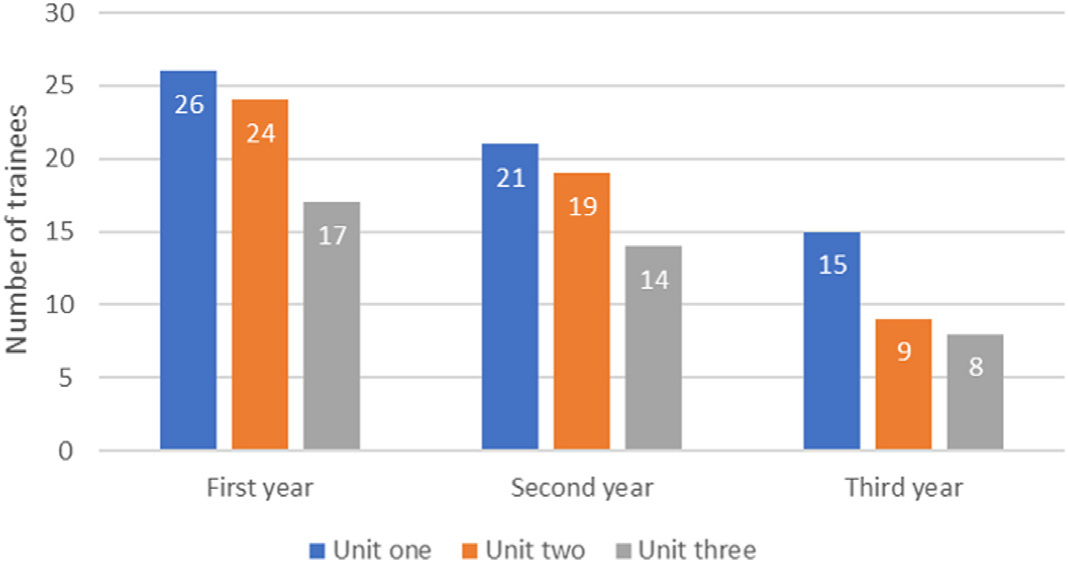
Demographics by Class Year

Figure 2 illustrates the number of residents in the curriculum by class year. The data show that first-year residents had the highest retention. All class years experienced a decrease in the number of residents over the 3 units, with the most significant drop occurring between unit 2 and unit 3. Proportionally, first-year residents had the smallest attrition (34.5%) by the start of the final unit, while third-year residents had the largest proportional attrition (46.7%).

**Figure 3:**
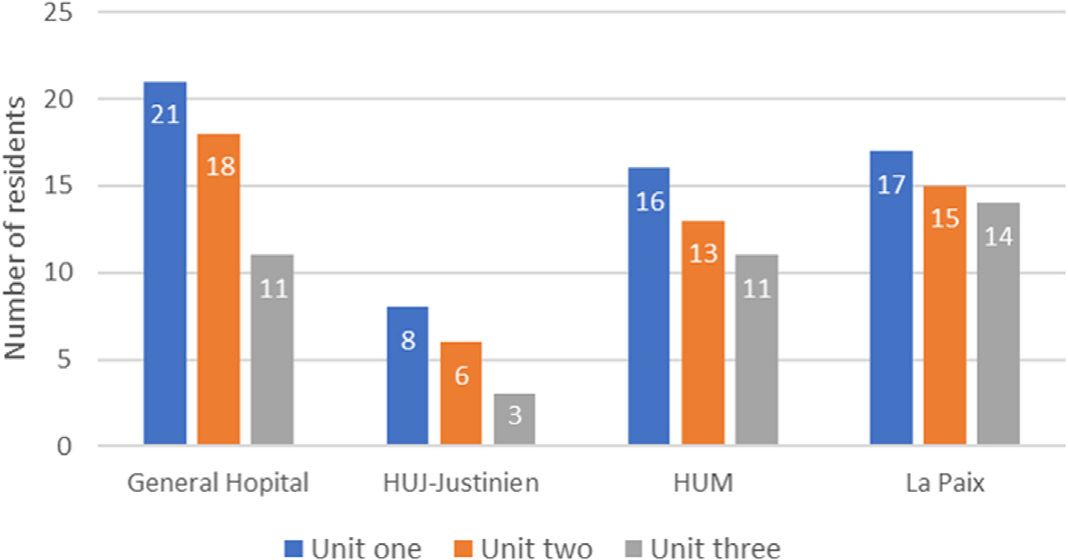
**Residents at Each Hospital** HUM = Hôpital Universitaire de Mirebalais; HUJ = Hôpital Universitaire Justinien; La Paix = Hôpital Universitaire de la Paix.

The number of residents at each hospital was measured at the start of each unit, revealing a decrease in participation over the course of the year across all hospitals. As the largest training program, General Hôpital had the absolute highest number while HUJ had the least. In contrast, HUJ had the highest attrition (62.5%), and La Paix had the lowest attrition rate (17.6%). There was a dedicated outreach mission to maintain engagement to La Paix whereas HUJ experienced a strike for resident salaries at the start of the program. These trends are illustrated in Figure 3.

**Figure 4:**
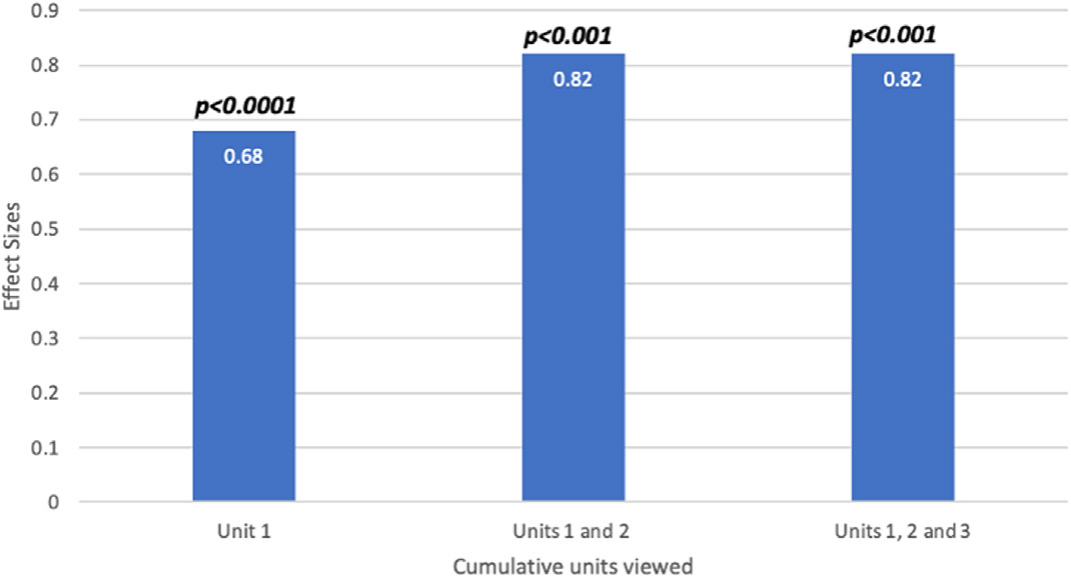
Effect Sizes by Cumulative Units for All Trainees

The effect sizes for all students were assessed, comparing those who participated in units 1 and 2 only versus those who completed all 3 units. Unit participation was defined as attendance of at least 80% of the coursework and assessments. There was a statistically significant large effect size for both the combined units 1 and 2 and all 3 units. Thirty-nine residents attended at least 80% of the lectures and completed all of the coursework and assessments for all 3 units. These findings are detailed in Figure 4.

**Figure 5:**
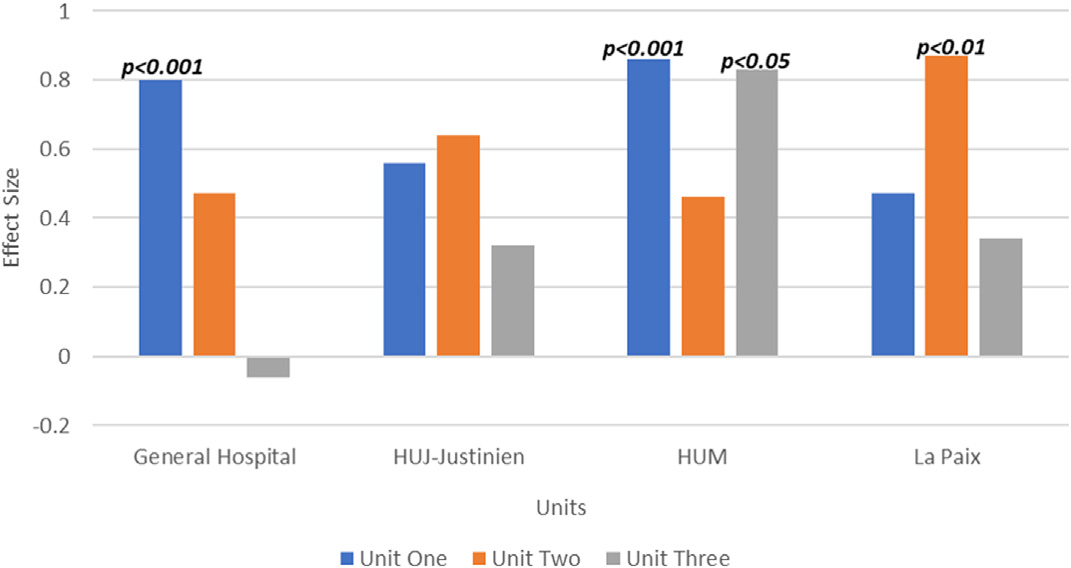
Effect Sizes by Hospital Abbreviations as in Figure 3.

The effect size by unit for all trainees from each specific hospital was assessed, revealing statistically significant increases. General Hôpital and HUM showed statistically significant increases in effect size in unit 1, La Paix in unit 2, and only HUM in unit 3. Across all 4 hospitals, the trend demonstrated moderate to large effect sizes, particularly in units 1 and 2. These results are detailed in Figure 5. General Hôpital demonstrated a statistically insignificant negative effect size in unit 3 representing no improvement in post-unit 3 assessment scores.

**Figure 6:**
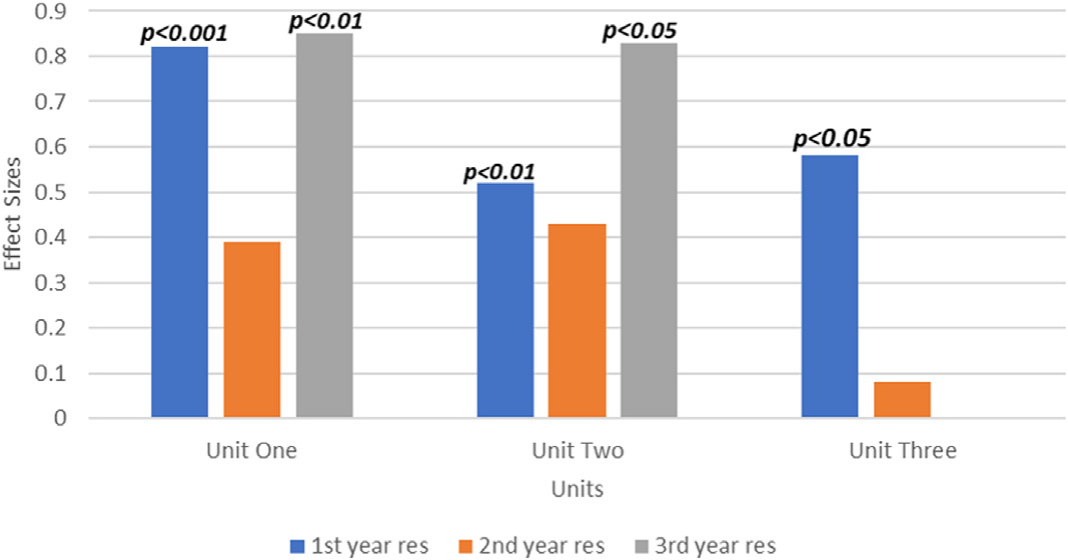
Effect Size by Class Year

The effect size for each unit by residency year is represented in Figure 6. First-year trainees exhibited a statistically significant large effect size (>0.5) across all units. Similarly, third-year trainees demonstrated a statistically significant large effect size in both units 1 and 2. However, as there was no change in the pretest and posttest scores for residents in unit 3, their effect size was effectively zero. Second-year residents demonstrated a trend toward positive effect sizes across all units (Central Illustration).

**Central Illustration:**
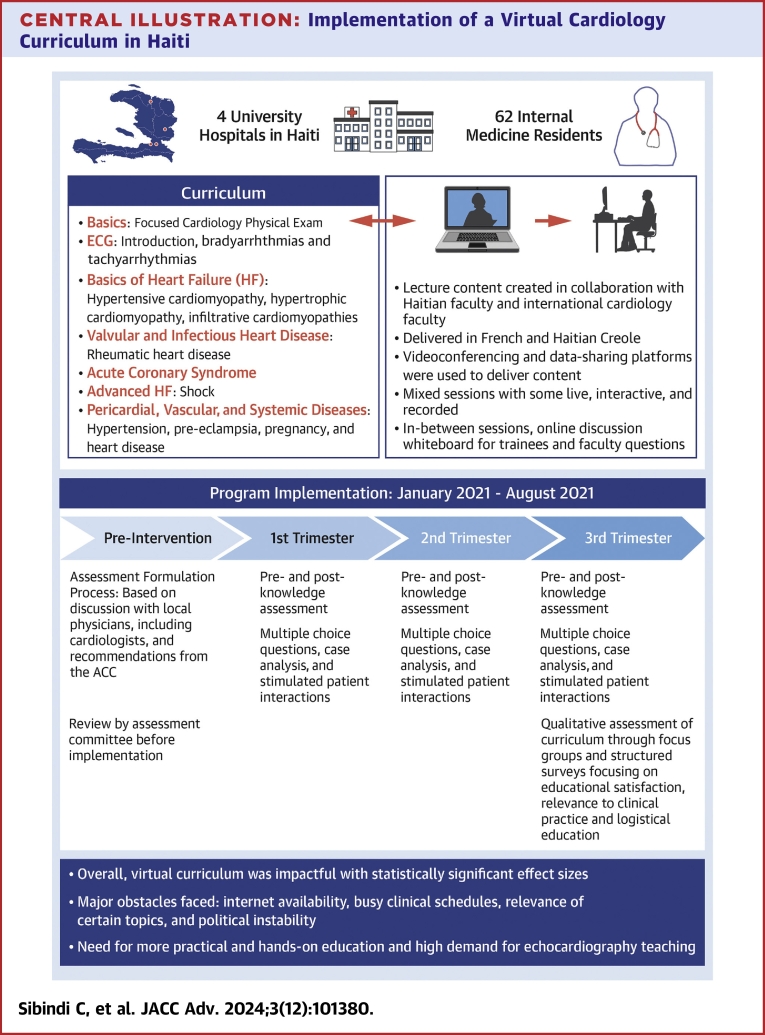
**Implementation of a Virtual Cardiology Curriculum in Haiti** ACC = American College of Cardiology; ECG = electrocardiogram.

## Discussion

Overall, our study demonstrates the feasibility of delivering cardiology education to internal medicine trainees across in LMICs like Haiti via a virtual, synchronous and asynchronous curriculum. This is contextualized as part of a larger goal of training nonspecialist clinicians in CVD to address the emerging global CVD epidemic. Our study sought to address this goal by delivering cardiology education on a virtual platform to medicine trainees in urban and rural communities across Haiti. Our analysis demonstrates that there was statistically significant knowledge acquisition as measured by the effect sizes (Figure 4) across all 3 units that covered a wide array of locally relevant cardiovascular topics (Supplemental Table 1). Additionally, there was notable knowledge acquisition across all 4 teaching hospitals. To our knowledge, this is the first national implementation of a virtual cardiology curriculum to address known cardiovascular educational deficits in Haiti.

In a prior study, we demonstrated that it is possible to deliver CVD education via an asynchronous virtual curriculum at a single center.^11^ This study is an extension of that pilot program. Expanding across an entire low-income nation provides unique challenges as it introduces increased heterogeneity. There is hospital geographic diversity which inherently brings a diversity of patients as well as patient pathology. For instance, General Hôpital is located in Port-au-Prince while HUM is located in Mirebalais, a more rural area 60 km outside of Port-au-Prince. The differences in geography can also lead to differences in internet connectivity. HUJ located in the north experienced significant internet disruptions as well as a salary-related strike among trainees during the study period. Figure 5 demonstrates that despite a wide array of challenges, however, there was a trend toward knowledge acquisition across all residency training programs with most having moderate to large effect sizes. This also demonstrates the advantage of a virtual curriculum which allows for the wide dissemination of information and through the virtual discussion board, the ability of clinicians to share cases, ideas, and expertise with colleagues across the country.^12^

When assessing performance based on residency year, we found that all 3 classes demonstrated a trend toward moderate to large effect sizes across unit 1 and unit 2, but only first and third year residents demonstrated statistically significant knowledge acquisition for those 2 units (Figure 6). Overall, the fact that first-year residents had the largest effect size can be explained by their expected lower baseline fund of knowledge compared to second- and third-year residents. This aligns with the concept of a ceiling effect in knowledge acquisition for third-year residents, as they were likely already familiar with some of the concepts presented in the curriculum. Nevertheless, all 3 classes demonstrated knowledge gains, as indicated by the positive effect sizes, which reflect the overall positive impact of the curriculum. Importantly, the same lectures were presented to trainees across all 3 years. Moving forward, this approach will be modified to provide tailored lectures with varying levels of complexity for each year, in order to prevent the ceiling effect and to optimize knowledge acquisition across all class levels.

Regarding the cardiology content delivered to trainees, most of the curriculum used was based on our previous work.^11^ This is because Haiti lacks a formalized national cardiology curriculum. The virtual program was developed with input from local Haitian clinicians, ensuring cultural sensitivity in addressing cardiovascular pathophysiology and management. This collaboration also highlighted unique resource constraints that would have been overlooked without local expertise.^11^ While the curriculum has enhanced trainee knowledge, there remains a need for a standardized national cardiology curriculum and defined training expectations for internal medicine trainees.

Many studies have demonstrated the efficacy and use of virtual curricula to deliver education to medical trainees.^12^^,^^13^ The recent COVID-19 pandemic significantly accelerated these efforts. Previous studies have highlighted various methods for delivering virtual curricula, including telemedicine, e-tutoring, and web-based learning. ^14^ Our project employed a custom-designed platform that integrated multiple modalities, such as prerecorded lectures, live sessions, and summative assessments, which likely enhanced the overall effectiveness of the curriculum. Additionally, our team solicited feedback on the platform from our participants and will be utilizing this to iterate the platform for improvement.^14^

There was notable student attrition in this study. Some attrition, up to about 20% was expected based on prior virtual education interventions.^15^ However, based on focus groups conducted with the trainees, we know that the political instability caused by the assassination of the Haitian president in 2021 had an impact on engagement.^14^ General Hôpital was disproportionately impacted by the political unrest due to its location in the middle of Port-au-Prince and because it is the largest public hospital in the country. The hospital suffered several shutdowns due to staff safety concerns. This likely explains the nonstatistically significant negative effect size for General Hôpital for unit 3. Attrition was also most pronounced among the third-year class which can be explained, in part, by the need to study for their capstone exams while also seeking employment opportunities. Another contributory factor was a resident strike at HUJ which limited engagement during the study period.^14^ Of note, there was also variable hospital leadership engagement among the training programs which also likely contributed to the varying resident engagement and attrition rates between hospitals. To improve attrition rates in the future, we plan to engage hospital leadership more proactively to enhance their involvement in the process, facilitating the seamless integration of the cardiovascular curriculum into the training program while further motivating resident participation. Lastly, while virtual platforms confer flexibility and dissemination, this can also inadvertently potentiate lack of engagement due to lack of in-person interaction.^16^ Such platforms thrive when embedded as part of a core curriculum which includes in-person or live teaching. As such, formalizing this program as a required component of the current internal medicine residency teaching programs with occasional in-person participation of local experts could strengthen the attendance and reduce attrition over time.^17^

There were some limitations and external factors that affected our study. Limitations of our work include internet connectivity and safety concerns which were particularly significant in the unit 3. This was because unit 3 took place in the setting of national instability given the assassination of the Haitian president and increased gang violence. This geopolitical issue also affected attendance of classes as well as the ability to reach a stable internet connection for the trainees. Such connectivity issues could be mitigated with internet stabilization through future financial investment. Another limitation was the small sample size which decreased further over the course of the study period due to the aforementioned reasons. Additionally, the absence of hands-on and in-person teaching, which is a centerpiece of most CVD education, is also notable. While the virtual curriculum demonstrated significant knowledge acquisition, the residents stated that the addition of hands-on and in-person teaching would enrich their learning experience especially for certain topics such as echocardiography.^14^^,^^18^ Lastly, while the residents were assessed after each unit which represented 3 to 4 months, retention over the longer term is an important aspect that will need to be studied. A comprehensive assessment, particularly as part of a formal curriculum, at the end of the year could represent a test of this longer-term knowledge retention.

## Conclusions

The cardiovascular burden in LMICs like Haiti, along with the pressing need to enhance cardiovascular education among nonspecialists to alleviate this burden, is evident. In this study, we demonstrated the feasibility of scaling and delivering a national virtual cardiology curriculum to medicine trainees across Haiti with significant knowledge acquisition measured by way of effect sizes. In terms of our future work, we are developing a focused cardiac ultrasound teaching intervention package to address the need for hands-on echocardiography skill development. We are also working with the Ministry of Health in Haiti to formalize cardiovascular education for nonspecialists through a locally recognized certification program.

Perspectives **COMPETENCY IN MEDICAL KNOWLEDGE:** Although this study did not concentrate on specific clinical practices, it offers an innovative approach to delivering cardiovascular education in LMICs. This approach is especially valuable in light of the recent COVID-19 pandemic, which limited the ability to conduct in-person training. Additionally, challenges such as political instability and time constraints can further hinder capacity-building efforts in LMICs. This model, however, enables the ongoing education of trainees despite these obstacles.

**TRANSLATIONAL OUTLOOK:** The cardiovascular knowledge trainees gained from this curriculum can be utilized clinically to better diagnose and treat patients with CVD in Haiti.

## Funding support and author disclosures

The authors have reported that they have no relationships relevant to the contents of this paper to disclose.
