# Supplementary figures and images for: Implementation of a Virtual Cardiology Curriculum to Address the Deficit of Cardiovascular Education in Haiti
Source: JACC Adv. 2024 Dec 26;3(12):101380. doi: 10.1016/j.jacadv.2024.101380 (PMC11734044; doi:10.1016/j.jacadv.2024.101380)

**Supplement 1:**


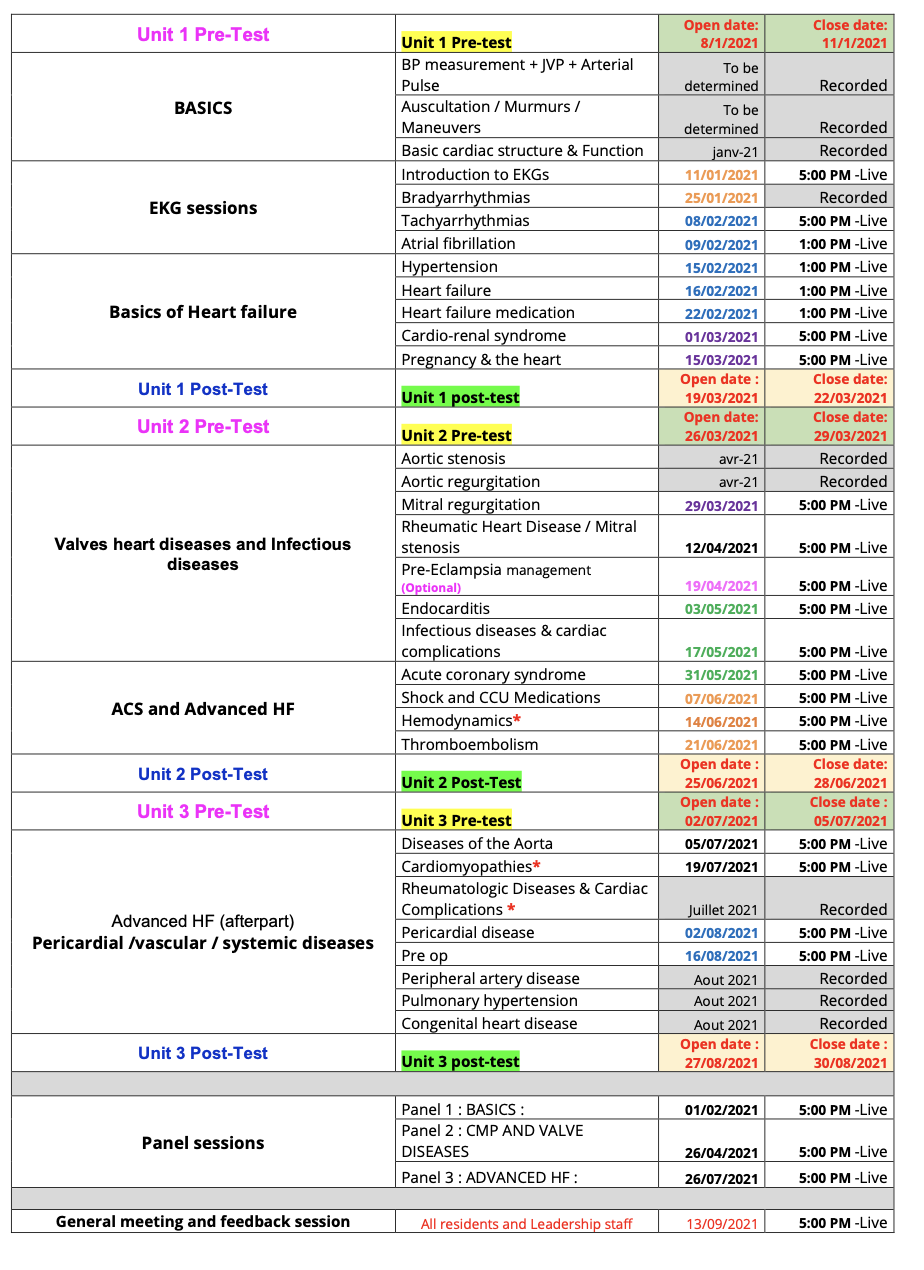

Supplement: Supplemental_Material [file mmc1.docx]
